# Exploring the promise of intersectionality for promoting justice-involved women’s health research and policy

**DOI:** 10.1186/s40352-020-00120-8

**Published:** 2020-07-25

**Authors:** Keren Gueta

**Affiliations:** Department of Criminology, Bar-Ilan University, 52900 Ramat-Gan, Israel

**Keywords:** Intersectionality, Justice-involved women, Health needs, Race, Class, Ageism

## Abstract

The perspective of intersectionality has gained widespread scholarly interest and been employed across many different disciplines, including criminology. This perspective focuses on interlocking systems of oppression and the need to work toward structural changes to promote social justice and equity. The present article aimed to explore the potential of intersectionality for advancing health research and policy regarding justice-involved women, in different phases of the judicial process, based on the extant literature.

First, employing an intersectional approach to analyze the issue of health during the pre-incarceration period may facilitate identification of the structural and representational factors underlying the barriers that women face in obtaining health services, which elevates the risk to their health. Furthermore, adopting an intersectionality perspective to explore women’s health during incarceration may shed light on vulnerable, invisible subpopulations of women such as incarcerated older women and their health problems, and help identify the structural barriers to carceral health services and the role of stigma in inflicting and normalizing harmful practices within prison walls. In addition, an intersectionality lens highlights the risk of unintended use of scholarly knowledge regarding the health of justice-involved women. Last, an intersectionality perspective is particularly relevant for research of the reentry of justice-involved women. In particular, it can be used to examine gender-sensitive reentry services that ignore other axes of marginalization, such as class and race, generating a powerful dynamic that results in partial service, denial of access to therapeutic resources, and possible exposure to health-damaging environments.

Through an exploration of the extant literature on justice-involved women, I endeavored to demonstrate that an intersectional framework offers powerful tools to both challenge and strengthen gender frameworks within criminology. This will make it possible to move beyond consideration of gender alone, to understand how systems of oppression based on race, age and other social locations intersect and combine to construct health disadvantages among justice-involved women. This highlights the needs for a new research agenda and policy that integrate the intersectional framework with health theories to provide a more developed understanding of health among justice-involved women.

## Introduction

Issues related to the health care of women in prisons have been largely overlooked in the research. In general, criminology literature has ignored the health effects of living in correctional facilities and the failure of services to meet the needs of justice-involved women (Bronson & Sufrin, 2019; Mignon, 2016). Nevertheless, scholars have shown that incarceration may exacerbate women’s health by adding more stress to their lives (Aday, Dye, & Kaiser, 2014), and limiting access to quality medical and dental care (Douglas, Plugge, & Fitzpatrick, 2009), leading to a call for greater consideration of gendered health factors.

However, although such scholarship on the health of justice-involved women is critically important to developing an understanding the role of gender, a focus on gender as the sole axis of marginalization is liable to obscure other axes that mediate the experience of justice-involved women (Bunn, 2018; Fader & Traylor, 2015) and limit the understanding of their health. More than a decade ago, Chesney-Lind (2006) called upon feminist criminologists to employ an intersectional perspective that considers multiple axes of marginalization that intersect with gender to shape the experience of justice-involved women. Potter (2013), who introduced intersectional criminology, noted the limited though growing body of scholarly literature in criminology that employs the intersectionality perspective, most of which focused on the lives of Black women in the United States and pathways to crime and incarceration. To the best of my knowledge, intersectionality theory has not been explicitly applied within a health context of justice-involved women.

The present article explores the merits of intersectionality in understanding the health needs and outcomes of justice-involved women. Drawing upon previous research findings, it suggests that a more nuanced understanding of the health of justice-involved women can be achieved by moving beyond a simple additive approach to social categories such as gender, race, class, sexuality, and age to develop recommendations for effective advancement of women’s health research and policy in the different stages of involvement with the justice system. To do so, it moves beyond gender analysis in pursuit of the possibilities offered by anti-essentialist feminist criminology.

## Justice-involved women: health risks and needs

In the United States, over 1 million women are involved in the criminal justice system – on probation, incarcerated, or on parole; they represent almost one-fifth of adults under correctional supervision nationwide (Kaeble, Glaze, Tsoutis, & Minton, 2015). Furthermore, during the last decade, the incarcerated female population has grown, outpacing that of incarcerated men. In this situation, it is particularly urgent to attend to the health needs of incarcerated women (Owen, Wells, & Pollock, 2017). In addition, in light of women’s pre-incarceration health risks and poor health practices that potentially jeopardize their health in prison, it is all the more important to attend to these women needs (Owen et al., 2017). Research has indicated that women enter prison with chronic health conditions and are at greater risk for behaviors such as prostitution, for sexually transmitted infections such as chlamydia, syphilis, HIV/AIDS, hepatitis C, as well as the human papillomavirus, which is associated with cervical cancer (Maruschak & Berzofsky, 2015). Furthermore, for women with trauma histories or prior mental health diagnoses, some aspects of incarceration may traumatize or promote suicidal ideation and behavior, ultimately worsening pre-existing mental health conditions (Harner & Riley, 2013). Last, specific challenges to women’s health within prison walls, such as pregnancy, intensify the need to understand and respond to their health needs (Bronson & Sufrin, 2019; Mignon, 2016).

Earlier feminist criminology often advocated the consideration of gender as a necessary step forward in understanding the needs of justice-involved women needs. However, there is now growing recognition that this perspective does not fully capture the experience of justice-involved women (Bell, 2017; Ocen, 2013). For example, the race-neutral framing of gender has been criticized as inadequate, especially given the evidence that women of color and indigenous women (e.g., African American, Latino, Māori, Native People, and Pacific Islanders) are incarcerated at significantly higher rates than any other group of women in their countries (Richie, 2012). Furthermore, incarcerated Black women are disproportionately impacted by health issues, including sexually transmitted infections, HIV/AIDS, substance use, and posttraumatic stress disorder (Binswanger, Redmond, Steiner, & Hicks, 2012; Hatcher, Toldson, Godette, & Richardson Jr, 2009). Nevertheless, although Black women represent the highest rate of incarceration among women, a recent review by Mahaffey and Stevens-Watkins (2016) indicated limited research of the significant disparities between the health of incarcerated Black women and that of Whites, Hispanics, and other racial/ethnic groups. This makes it difficult to measure the actual breadth of racial disparities and subsequently, to recognize the importance of identifying strategies to reduce them.

Thus there is a need for a perspective that promotes the health of justice-involved women by enabling scholarly analyses of how categories of race, gender, ethnicity, sexuality, age and geographical location affect women’s health. The results of such a body of research would inform and advance a gender perspective within criminology that is attuned to how interlocking systems of power operate and come to bear on notions of health.

## Intersectionality and intersectional criminology

Rooted in Black feminist thought and scholar-activists and introduced by Kimberlé Crenshaw (1991) over 30 years ago, intersectionality has advanced feminist studies in different areas beyond a gendered perspective of women’s experience or that including only gender and race. It has gained widespread appeal and been employed across numerous disciplines, to create a broad field of intersectionality studies (Cho, Crenshaw, & McCall, 2013). The perspective of intersectionality recognizes the significance of gender but does not consider it the most or the only important axis of experience, since this is liable to impair understanding. For instance, such a narrow view might frame discriminatory practices as suffered by racialized men and White women, rendering inequalities experienced by racialized women invisible (Crenshaw, 2011). Furthermore, intersectionality sheds light on the intertwined nature of different types of social inequality and power. Collins (2015) defined it as “the critical insight that race, class, gender, sexuality, ethnicity, nation, ability, and age operate not as unitary, mutually exclusive entities, but as reciprocally constructing phenomena that in turn shape complex social inequalities” (p. 2). For example, a newly diagnosed low income cancer patient may spend his/her few life savings on treatments. This process can limit his ability to benefit from treatments and will push the individual further to a marginalized geographical location with limited access to health care services and basic needs (e.g., affordable housing) (Baah, Teitelman, & Riegel, 2019).

Crenshaw (1991) charted three foci of intersection as analytical guides for assessment of this interplay. The first, structural intersectionality, enables the identification of socio-structural elements such as poverty and institutions that place women of color at a disadvantage. Thus, embracing intersectionality can facilitate a crucial shift in focus from the implied deficiency of individuals grounded in their multiple needs to the shortcomings of the responses by the systems responsible for addressing these needs (Bunn, 2018). The second, political intersectionality, addresses the discourses embedded in law, policies, social services, and even “objective” academic knowledge regarding women’s problems, such as domestic violence, that effectively silence or erase the experiences of women of color. This might be the result, for instance, in normalizing women’s problems by prioritizing White women’s experiences as victims, or by focusing struggles for social justice on sexism and not racism (Cho et al., 2013). Finally, representational intersectionality analyzes the broader cultural discourses to show how racist and sexist representations of women of color serve to further perpetuate and marginalize them (Crenshaw, 1991). For example, over the past five decades, the social construction of the “welfare queen” that highlights a specific subgroup of welfare beneficiaries as making illegitimate claims for government assistance, has been a consistent feature of calls for welfare reform (Cassese & Barnes, 2019; Collins, 2015).

Criminologists have long been interested in the relationship of crime with race, gender, and class, particularly in theories of women’s criminality. This understanding of intersecting identities that emerge from different systems of oppression has guided a focus on understanding the overlapping and cumulative effects of the intersections of multiple oppressions that affect women’s decisions to engage in crime (Brown, 2010; Burgess-Proctor, 2006). Furthermore, in her review of a substantial expanding body of criminological research that utilizes intersectionality, Potter (2013) found that intersectionality provides “a critical refection on the impact of interconnected identities and statuses of individuals and groups in relation to their experiences with crime, the social control of crime, and any crime-related issues,” and argued that this perspective is a “necessary evolution in criminological theory” (Potter, 2013, p. 305, 306). Ocen (2013) concluded that much of the intersectional criminological scholarship has detected trends and experiences of punishment that are often unaccounted for in criminology research. It has focused on documenting disparities in different phases of the judicial process (e.g. arrest, sentencing, incarceration, and parole) and how power operates via justice systems. Thus it deals with multiplicative axes of marginalization among women, low-income individuals, and communities of color that face harsher penalties, such as racial profiling and specific pathways to crime and imprisonment, and the devastating economic effects of involvement in the criminal justice system (see, e.g., Erez & Berko, 2010; Spohn & Sample, 2013). However, Potter (2013) called for “expanded use” of intersectionality “in all forms of inquiry and most certainly in any form of criminology that designates itself as critical” (p. 316). Furthermore, very few have theorized about the intersections of these factors and crime in substantively shaping the health of justice-involved women. As intersectional criminology has, for the most part, not yet been applied in the context of health, there is still limited insight regarding the structural barriers to health, intra-group differences in the experience of health disparities and vulnerability, and they ways they contribute to materialization of divergent health realities among justice-involved women.

Research on health in other disciplines offers knowledge of intersectionality that can benefit the investigation of health among justice-involved women. Intersectional research and theory enable elucidation of the social determinants of health by interpreting multiple and intersecting systems of oppression and privilege and interrelated sociocultural systems of power that shape people’s opportunities in life. These occur not only through deliberate political and structural oppression, but in a subtle, representational discriminatory fashion, as well (Hankivsky & Christoffersen, 2008). For example, guided by intersectionality theory, Baah et al. (2019) invoked the concept of marginalization as a process through which certain population groups experience multiple social determinants that concurrently limit their access to health-promoting resources and increase their risk for poor health. The authors first discussed health disparities and inequity in access to resources, due to an uneven distribution of political and socioeconomic resources across gender, race, sexual orientation, culture, and geographic regions that resulted in limited employment and educational opportunities, as well as affordable health care services. They went on to describe a situation of exposure and lack of protection against a health-damaging environment, due to interaction of the sociopolitical, economic, structural, cultural, and interpersonal circumstances that pose both physiological and psychological threats individuals. Finally, Baah et al. argued that marginalized individuals experience differential health care treatment based on acts of rejection that are perpetuated through ideologies such as racism, classism, and constrictive gender role norms and mechanisms such as implicit bias, bullying, mass incarceration, and disparities in unemployment rates, which lead to limited use of social and health care services.

## An intersectional perspective on the health issues of justice-involved women

### Women’s pre-incarceration health: barriers to healthcare services

The poor physical and mental health of justice-involved women is shaped by pre-incarceration health risks. Worldwide, studies have shown that the majority of women enter correctional institutions with complex mental and physical health issues, sexual and physical abuse, addictions, trauma, and violence exposure (Chen & Gueta, 2015; Covington, 2014). Women entering the justice system are at high risk for sexually transmitted infections for HIV/AIDS; hepatitis C; as well as the human papillomavirus, which is associated with cervical cancer; and chlamydia, gonorrhea, and syphilis, which are the associated with sexual victimization and prostitution (van den Beret al., 2010). However, for many justice-involved women, prison is the first setting in which they receive systematic medical care (Douglas et al., 2009; Fearn & Parker, 2005).

Adopting an intersectional perspective enables identification of the structural and representational factors underlying women’s barriers to receiving health services and draws attention to violence inflicted by the state, which in turn elevates the risk to their health. First, this perspective directs more attention to the structural forces that shape women’s health, rather than primarily addressing perceived individual failures that lead to poor health outcomes (Hannah-Moffat, 2006; Mahaffey & Stevens-Watkins, 2016). Accordingly, research has shown that many incarcerated women did not have access to health care services prior to imprisonment, due to structural barriers related to class (Mignon, 2016; Rich, Cortina, Uvin, & Dumont, 2013). The literature has indicated barriers to treatment that are shaped by structural inequalities associated with poverty, racism, housing insecurity, and unemployment among women (Grella, 2008).

Furthermore, by urging recognition of differences among women rather than a monolithic gender-based view, an intersectional perspective enables the identification of particularly vulnerable populations (Collins, 2015). Thus, for instance, Black female inmates may constitute a special group with particularly limited access to health insurance prior to incarceration (Bonney, Clarke, Simmons, Rose, & Rich, 2008; Mahaffey & Stevens-Watkins, 2016).

Another vulnerable population among justice-involved women is that of mothers. Motherhood can be a barrier to seeking health services, due to the fear of losing custody. For instance, despite the motivation of justice-involved women to stop using drugs for the sake of their future health and their children’s welfare (Moe, 2006), the fear of losing children upon revealing addiction can pose a major barrier to seeking treatment and might limit the use of social services, despite awareness of the need for them (Gueta, 2017). Motherhood could also be a barrier to seeking health services, due to gender-related characteristics of treatment services that ignore women’s needs such as child care (Grella, 2008) or their poor mental and physical state after giving birth (Gueta, 2017).

Furthermore, according to the representational intersectionality perspective, race and class intersect to create distinctive experiences of motherhood (Collins, 2015); this underscores how motherhood can be a barrier to health services for low-income and minority women. Accordingly, the discourses of good and bad motherhood reproduce the ideological power of personal responsibility, thus precluding an image of a deserving low-income mother or black women (SmithBattle, 2007). Given the severe social stigma surrounding mothering and drug use (Campbell, 2000), research has shown that children of minority and low socio-economic groups were more likely to be assessed as being at risk and more likely to be removed from their homes than those of higher socioeconomic status (Enosh & Bayer-Topilsky, 2015). Thus, the threat of the removal of children from their care is more pervasive and substantial for these mothers and discourages them from accessing social and health services. Furthermore, the discourse of bad motherhood is grounded in identification of poverty with individual failure. Its association with race and class ideologies has also played a pivotal role in limiting public policies, medical practices, social services, and benefits for mothers living in poverty, and social marginalization exacerbates their stress and limits their access to social and health care (SmithBattle, 2007). In addition, gender-related characteristics of treatment services that ignore women’s needs such as child care are particularly problematic for low-income and minority women (Grella, 2008). As a result, the challenges of mothering are particularly onerous for economically disadvantaged and socially isolated women, who are at greater risk of the devastating results associated with loss of child custody, which has been conceptualized as trauma at the hands of state institutions (Kenny, Barrington, & Green, 2015).

According to Bowleg (2012), a critical intersectional approach that focuses on the dynamic between gender and structural inequities to tackle the above health-risk determiners could inform research and policymaking to improve health services for women. The above analysis demonstrates how the intersectional perspective can guide a shift in policy and practice that brings social factors to the foreground, to establish and facilitate health care and reject drug policies that punish people of minority and low economic status (Gueta, 2017). Specifically, Rich et al. (2013) recommended two ways that the Affordable Care Act provides an opportunity to address the pressing health needs of justice-involved women. First, it promises to improve access to health care by reducing financial barriers to care for women prisoners and second, it offers expanded coverage of behavioral health care related to treatment for mental illness and/or illicit substance use. Furthermore, a holistic intervention that recognizes that children could constitute a vital resource of support in the struggle with psychological and symbolic aspects of poverty, especially for women of color, may be another important step toward this direction (Owen et al., 2017).

### Women’s health during incarceration from an intersectional perspective

The pre-incarceration state of health of justice-involved women is further complicated by the carceral environment. First, two-thirds of incarcerated women are mothers to minor children (Glaze & Maruschak, 2008), and this may have unique implications for their health needs. Furthermore, although nearly three-quarters of incarcerated women are aged 18–44, the prime childbearing years (Glaze & Maruschak, 2008), the results of a recent systematic review suggest that mental health among pregnant prisoners is a major concern that has not been adequately addressed (Bronson & Sufrin, 2019).

Second, the limited access and quality of health services in prisons contributes to a wide variety of unfavorable health outcomes among women (Allen, Flaherty, & Ely, 2010; Harner & Riley, 2013). Finally, certain aspects of the carceral environment, such as vulnerability to violence and the experience of being detained and isolated from one’s family, may have specific implications for justice-involved women’s health (Travis & Waul, 2003).

Moreover, utilizing an intersectionality perspective to explore the health of women during incarceration may help uncover a vulnerable, hidden subpopulation of women and their health problems, identify structural barriers for health, shed light on the role of stigma in inflicting and normalizing harmful practices, and help detect the potential for abuse of scholarly knowledge regarding women’s health. Embracing an intersectionality perspective that rejects a monolithic view of the health experience of justice-involved women could highlight a hidden subpopulation of women and their health problems. There is a paucity of research literature on the health issues of incarcerated older women. Among this group, those coping with terminal illness while in prison present unique health challenges to correctional healthcare providers. In addition, older women often suffer from chronic mental and physical health conditions such as arthritis, hepatitis, and heart conditions (Aday & Krabill, 2011; Handtke, Bretschneider, Elger, & Wangmo, 2015). The literature on elderly justice-involved women indicates specific health-risk factors, such as uncaring attitudes of medical staff towards them and lack of trust in prison healthcare providers, which create barriers to receiving health care (Aday & Farney, 2014), or violence against elderly women by younger inmates (Aday & Krabill, 2011). These findings, along with the documented successful aging process among older Filipino prisoners, serve as an impetus for structural and procedural changes in prison, with a view to providing an environment that promotes well-being and successful aging among older inmates (Lucas, Lozano, Valdez, Manzarate, & Lumawag, 2018).

Furthermore, although sexual victimization and its associated harmful impact on physical and mental health threaten all justice-involved women, as one-quarter of incarcerated women are sexually abused (Mardorossian, 2012), employing an intersectionality perspective may shift the focus toward especially vulnerable justice-involved women. For example, sexual orientation and racial background have been shown to increase vulnerability for sexual victimization. There is growing evidence of higher victimization rates among gay and bisexual inmates relative to the prison population as a whole (Hensley, Castle, & Tewksbury, 2003; Struckman-Johnson & Struckman-Johnson, 2006), but the victimization of transgender women while incarcerated, and associations of transgender status with health have gained very limited research attention. One exception is a study by Reisner, Bailey, and Sevelius (2014), who analyzed data from the National Transgender Discrimination Survey, a convenience sample of transgender adults in the United States (*n* = 3878). The findings show that Black and Native American/Alaskan Native transgender women were more likely to report a history of incarceration than White (non-Hispanic) respondents were, and those with a history of incarceration were more likely to report negative health-related indicators, including self-reports of being HIV-positive.

Ocen (2013) analyzed feminist legal scholarship discourse on the issue of sexual victimization of incarcerated women by male prison guards from an intersectional perspective. The findings indicated a focus of this body of research on sexual assault as a mechanism to maintain dominance and control over women. However, Ocen (2013) indicts that the research has largely ignored the subject of sexual abuse of Black women as an expression of racial dominance and how constructions of Black women as sexually available influence the forms of violence imposed upon women prisoners. This indicates the need for examination of additional risks factors for sexual victimization and a balance between the desire to protect privacy in prisons and the importance of finding new ways to tackle sexual victimization among female inmates, such as technological surveillance (Hensley et al., 2003; Reisner et al., 2014).

In addition, intersectional analysis directs attention to the structural factors, particularly associated with criminal justice healthcare services, that underpin women’s health during incarceration. For instance, research from this perspective has shed light on the role of co-payment. In about 70% of United State prisons, prisoners are charged a fee of between $2 and $10 for each request for health care, and this has been shown to reduce access to health care and lead to poor health outcomes (Fisher & Hatton, 2010). In particular, the practice of co-payment has been found to affect inmates such as older women, who have multiple medical concerns but delay seeking necessary treatment because of the financial burden (Fisher & Hatton, 2010).

Another example of structural barriers grounded in correctional health services may be the common penal policy that is based on women’s pathways to crimes, whereby illness and trauma shape gender-responsive treatment (Covington, 2014). Intersectionality highlights the potential implications for some women of this monolithic, uniform approach. Correctional mental health policies and programs informed by trauma and illness-based approaches might foster individualized and “pathologized” understandings of female offenders, thus separating the experience of justice-involved women from the political construction of their offending behavior (Hannah-Moffat, 2006; Kilty, 2012; Pollack, 2007). Furthermore, in an analysis of the Offender Personality Disorder program, which was designed to address women’s unmet mental health needs, Player (2017) showed how even a well-intended gender-responsive program can inflict harmful consequences on women, especially women of color, and might expand the disciplinary and regulatory powers of the prison. According to Player (2017), the focus on the clinical link between the women’s offending behavior and their personality disorder, as reflected in that program, obscured the intersectional dynamics of gender with race and ethnicity, as well as other sources of inequality, by translating their experiences of victimization and therapeutic needs into correctional risk factors. For example, Player indicate that imprisoned woman that receive the psychiatric diagnosis of borderline personality disorder, as part of her participation in the Offender Personality Disorder program, may be subjected to expanded period of correctional supervision. Furthermore, this diagnosis may shape her release program obscuring the structural contexts such as poverty that contributed to her offending.

It seems that representational intersectionality could have powerful effects on health, especially if it indicates stigma, and this might provide criminologists with another mechanism for understanding health disparities. Specifically, this perspective directs attention to the idealized forms of femininity, such as motherhood and stereotypes and their impact on the health of justice-involved women. Incarceration puts all mothers’ rights and images at risk, since they are often portrayed as “inadequate, incompetent mothers who are unable to provide adequately for the needs of their children” (Travis & Waul, 2003, p. 76), and this could harm their mental health. However, these perceptions may be intensified in the case of women of color and women who are viewed as not conforming to gender roles. This could even lead to harmful practices, such as forced medical sterilization to punish those who are constructed as “deviant” and therefore undeserving of being mothers (Ocen, 2012, 2013). In addition, Ocen (2012) noted that although the practice of shackling women during pregnancy and even childbirth, which was employed in at least 34 states across the United States, was criticized and subsumed under the overall rubric of gender, this practice was normalized and justified within institutions with regard to Black justice-involved women by constructing them as masculine, deviant, and dangerous.

Last, the intersectional perspective urges criminologists to ensure that the knowledge they generate will not contribute to more criminalization and legislation that supports more widespread incarceration (Henne & Troshynski, 2019). For example, the findings of improved mental and psychological health of women during incarceration due to access to psychotropic medication in prison, participation in mental health counseling, avoiding exposure to violence, and women’s perceptions of incarceration as a means to access health care services (Douglas et al., 2009; Harner & Riley, 2013) could be abused by increased reliance on incarceration as a mechanism for responding to women’s health needs. In contrast, these findings should be interpreted as indications of the alarming status of women’s health and safety in the United States and worldwide. Furthermore, it is important to bear in mind that state interventions invite the possibility of reinforced intersectional inequalities, even when presented as responding to intersectional demands (Henne & Troshynski, 2019).

### Women health issue during reentry

Research has shown that compared with the general public, released inmates are at high risk for physical and behavioral health morbidity and mortality mainly due to drug overdose, followed by cardiovascular disease, homicide, and suicide, which are often correlated with a low quality of physical health, chronic disease, ongoing trauma, and stigma (Binswanger et al., 2011; Honorato et al., 2016; Moore et al., 2016). Moreover, the literature suggests that reentry needs may differ by gender, given the higher rates and/or greater severity of many mental health problems among women, compared with men, the impact of imprisonment itself on women’s health, and their mothering role (Owen et al., 2017). Given the findings that receiving mental health treatment during reentry is associated with lower reincarceration rates (Brown, Hickey, & Buck, 2013), the lack and discontinuity of mental health services becomes alarmingly detrimental to women health at reentry (Baillargeon, Hoge, & Penn, 2010).

While gender scholarship is critically important in developing the understanding of the role of gender, the use of gender as the main factor in examining the reentry experience may obscure the role of other discrimination and marginalization factors in mediating—and often exacerbating—the reentry experience (Bunn, 2018; Fader & Traylor, 2015). The praxis of intersectionality becomes palpable in the therapeutic discourse, which obscures the realities of past and/or current victimization that are shaped not only by gender, but also by racial and class inequalities, because it individualizes women’s problems and resolutions and attributes them to women’s “disorderly lives” (Hackett, 2013, p. 224). By the same token, race-neutral services may be partial services. For example, Brown (2010) indicated that although the reentry challenges of many justice-involved women is to reunite with their children, African-American women bear an extra burden, compared to white and Latino women, due to mass incarceration of African-American men, which limits the support for childrearing. Reentry services that do not address these additional complexities for African American women increase their vulnerability to recidivism and health problems. In addition, during the reentry phase, women are at particularly high risk for unplanned pregnancies, a significant and costly public health problem, in light of the high rates of poverty, substance abuse, and sexually transmitted infections in this population. However, this lacuna in the knowledge and practice is even more alarming given the disproportionate incidence of unintended pregnancy, inadequate access to prenatal care, maternal mortality, and health complications due to pregnancy and its termination among women of color (Roth, 2010).

In addition, intersectionality theory is particularly relevant for reentry research, because it shows how developing reentry services based on gender needs alone, ignoring how oppression based on other axes such as class and immigration has produced a powerful dynamic, whereby women are denied access to therapeutic resources. For example, Begun, Early, and Hodge (2016) found different financial barriers to receiving substance use services and mental health care among men and women during community reentry following incarceration. Furthermore, gender-sensitive reentry programs must involve health and housing solutions to reduce addiction, recidivism, and poor health among women without fear of losing custody of children and not assuming that “the role of motherhood as a conventional identity and script for reform” is possible without financial formal support (Brown & Bloom, 2009, p. 332). Bunn (2018) also highlighted the role of structural barriers and the quality of services available to ex-prisoners upon release. She argued that the nature of “needs” within the returning prisoner population is not simply multiple, but rather intersectional, showing how multiple needs such as substance abuse and mental illness interconnect with disability, class, gender, and numerous other categories to produce unique experiences of marginalization due to exclusion from one system. For instance, post-release support services (e.g., drug treatment services) facilitate the exclusion of ex-prisoners from other services (e.g., the disability service system), thereby rendering their intersecting needs invisible, and, in turn, escalating their health needs.

Last, research has indicated that limited access to affordable housing is the most acute problem for women, because it forces them to return to living environments in which they are at risk of violence (Bunn, 2018; Owen et al., 2017). This demonstrates the role of structural factors in partial reentry services, in which women are exposed to an unprotected environment where the interaction of sociopolitical, economic, structural, cultural, and interpersonal circumstances put them at physiological and psychological risk (Baah et al., 2019). In an exploratory study of the effects of such health-damaging environments conducted among 204 women in Kansas City jails, Ramaswamy, Kelly, Koblitz, Kimminau, and Engelman (2011) revealed an association between experiences of violence and incarcerated women’s self-reports of cervical cancer screening and cancer history and treatment. The findings show that participants who did not fear neighborhood violence were less likely to report an abnormal Pap history and more likely to visit a family doctor for their Pap screenings.

## Conclusions

In this article, I used an intersectional perspective as a conceptual lens to demonstrate its transformative potential for understanding the health of justice-involved women. Through an exploration of gender research, I endeavored to demonstrate that an intersectional framework offers powerful tools to both challenge and strengthen gender frameworks within criminology. This will make it possible to move beyond consideration of gender alone, to understand how systems of oppression based on race, class, age, and other social locations intersect and combine to construct health disadvantages among justice-involved women.

The call for an intersectional approach to the subject of the health of justice-involved women is more than just a theoretical exercise of integration. It could affect the practice regarding this life-and-death issue since the position of women of color in lower social strata put them at higher risk of death due to health problems associated with criminal justice involvement compared with White women (Mahaffey & Stevens-Watkins, 2016).

The importance of this intersectional model for feminist criminology is underscored by this discussion of implications and recommendations for theoretical praxis, policy, and programs regarding the health of justice-involved women. First, the transformative promise of the intersectionality perspective to the understanding of the health of justice-involved women lies in its non-essentialist understanding of women’s health in general. Its focus on the dynamic of multiple axes of discrimination and privilege (Hankivsky et al., 2010) makes health risks for justice-involved women, such as age and sexual orientation, visible. Furthermore, an intersectional approach demands further research on the health needs of subpopulation of women such as asylum seekers. The intersection between immigration and criminalisation is not often the focus of academic literature in this field despite that race, ethnicity, nationality and citizenship shape border practice (Parmar, 2018).

Second, another transformative promise of the intersectional framework for understanding the health of justice-involved women lies in the shift of focus from individual deficiencies, which is prevalent in criminology theories on this issue, to the oppressive processes of control of women by the criminal justice, health, and welfare systems (Collins, 2015; Ocen, 2012). This, in turn, indicates the need for a power shift in the development of social services programs toward greater user cooperation and more inclusive models and programs. Instead of concentrating on pathologies and responses of women, intersectionality draws attention to the structural forces and cultural discourses that intersect and shape the health status of justice-involved women inside and outside prison walls. Furthermore, given the specific challenge for many criminologists to address the structural violence enacted by the state (Henne & Troshynski, 2019), integration of an intersectional perspective for scholarly insights regarding the health of justice-involved women may provide an opportunity to generate more effective strategies to combat the health risks created by mechanisms of social control, such as the practice of shackling women during pregnancy and childbirth (Ocen, 2012).

To conclude, an intersectional approach to the study of health of justice-involved women health can generate a non-monolithic understanding of women’s health, place greater emphasis on the structural underpinnings of women’s health problems, shed light on women’s use of incarceration as a means to access health care services, and explore the health implications of representations of incarcerated women in the context of other axes of marginalization.

## Data Availability

Not applicable- the paper is based on a critical literature review.
